# Supplementary material for: Modelling Hyperglycaemia in an Epithelial Membrane Model: Biophysical Characterisation
Source: Biomolecules. 2022 Oct 21;12(10):1534. doi: 10.3390/biom12101534 (PMC9599690; doi:10.3390/biom12101534)
Supplement: Supplementary file 1 [file biomolecules-12-01534-s001.zip › biomolecules-1966120-supplementary materials.pdf]

Supplementary Material

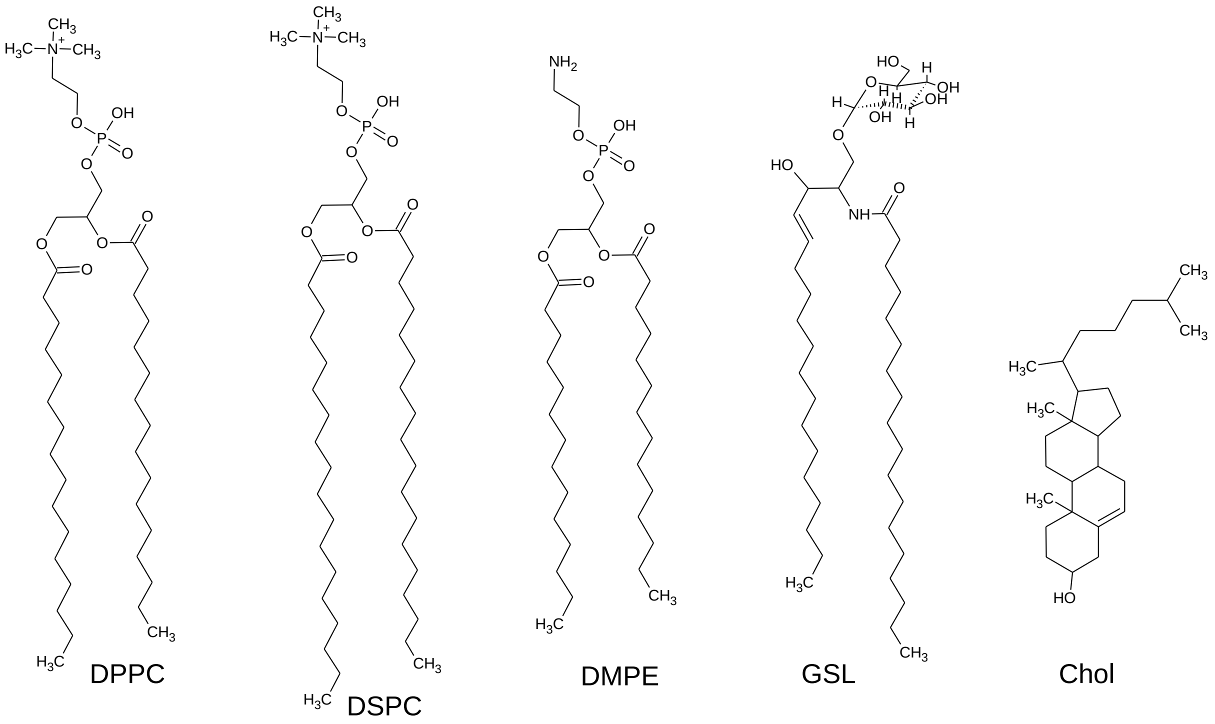

Supplemental Figure S1. Structure of lipids included in the membrane model. DPPC: di-palmitoyl-glycerophosphatidylcholine; DSPC:di-stearoyl-glycerophosphatidylcholine; DMPE: di-myristoyl-glycerophosphatidylethanolamine; GSL: glucosyl-sphingolipid; Chol: cholesterol.

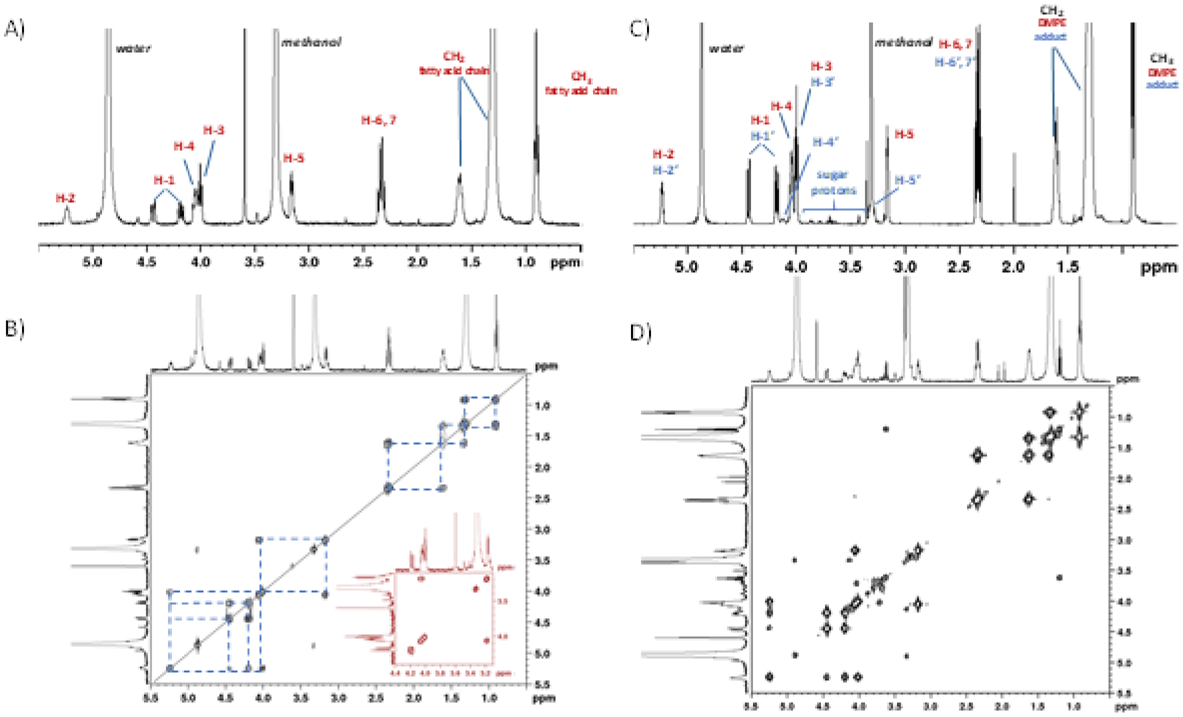

Supplemental Figure S2. ^{1}H NMR (A) and $^{1}H/^{1}H$ COSY (B) spectra of unmodified DMPE in CD_3OD, and ^{1}H NMR (C) and $^{1}H/^{1}H$ COSY (D) spectra of the DMPE-glyc mixture in CD_3OD.

^{1}H NMR spectrum description: The ^{1}H NMR (600.13 MHz, CD_3OD) for the reaction mixture shows signals at δ= 0.90 (t, J= 7.0 Hz, CH_3 from DMPE and adduct), 1.26-1.34 (m, CH_2 from DMPE and adduct), 1.58-1.62 (m, CH_2 from DMPE and adduct), 2.32 and 2.34 (2t, J= 7.2 Hz, H-6, 7 from DMPE and H-6', 7' from adduct), 3.16 (t, J= 4.8 Hz, integrate to 2.00 protons, H-5 from DMPE), 3.26-3.28 (m, H-5' from adduct), 3.60-4.00 (several m, sugar residue from adduct), 4.00 (t, J= 5.9 Hz, H-3 from DMPE and H-3' from adduct), 4.03-4.06 (m, H-4 from DMPE), 4.11-4.14 (m, integrate to 0.0924 protons, H-4' from adduct), 4.18 (dd, J= 6.8 and 12 Hz, H-1 from DMPE and H-1' from adduct), 4.44 (dd, J= 3.2 and 12 Hz, H-1 from DMPE and H-1' from adduct), 5.22-5.24 (m, H-2 from DMPE and H-2' from adduct) ppm.
